# Impact of age, ethnicity, sex and prior infection status on immunogenicity following a single dose of the BNT162b2 mRNA COVID-19 vaccine: real-world evidence from healthcare workers, Israel, December 2020 to January 2021

**DOI:** 10.2807/1560-7917.ES.2021.26.6.2100096

**Published:** 2021-02-11

**Authors:** Kamal Abu Jabal, Hila Ben-Amram, Karine Beiruti, Yunis Batheesh, Christian Sussan, Salman Zarka, Michael Edelstein

**Affiliations:** 1 Ziv Medical Centre, Safed, Israel; 2 These authors contributed equally to this article and share first authorship; 3 Azrieli Faculty of Medicine, Bar-Ilan University, Safed, Israel

**Keywords:** COVID-19, vaccine, immunogenicity, Israel

## Abstract

The BNT162b2 mRNA COVID-19 vaccine showed high efficacy in clinical trials but observational data from populations not included in trials are needed. We describe immunogenicity 21 days post-dose 1 among 514 Israeli healthcare workers by age, ethnicity, sex and prior COVID-19 infection. Immunogenicity was similar by ethnicity and sex but decreased with age. Those with prior infection had antibody titres one magnitude order higher than naïve individuals regardless of the presence of detectable IgG antibodies pre-vaccination.

The coronavirus disease (COVID-19) pandemic, which started in 2019 in China, continues to spread, despite multiple lockdowns and prolonged control measures implemented in most countries. As at 25 January 2021, over 99 million cases and 2.1 million deaths were reported globally [1]. In December 2020, several vaccine candidates were shown to be safe and efficacious in trials [2-4] and mass vaccination (in combination with existing control measures) is seen as one of the central elements to controlling the pandemic. Although clinical trial data are encouraging, real-world evidence with regards to the vaccines remains scarce. In particular, describing immunogenicity and effectiveness among specific ethnic groups is important as the disease disproportionately affects certain ethnic minorities for reasons not fully understood but not fully attributable to socio-demographic factors [5,6]. Likewise, the post-vaccination immune response among those previously infected remains unclear. Here we present early results from vaccination in healthcare workers (HCWs) in Israel.

## COVID-19 vaccination in Israel

As at 25 January 2021, Israel had vaccinated 29.2% of its population with a single dose of vaccine [7], the highest proportion in the world, almost exclusively with the BNT162b2 mRNA COVID-19 vaccine (Pfizer, New York, United States (US) and BioNTech, Mainz, Germany). HCWs were among the first to be eligible.

Ziv Medical Center (ZMC), located in Safed, Israel, is a 350-bed hospital serving the north of Israel. It is staffed by a multi-ethnic workforce of ca 1,500 persons including Jews, Arabs and Druze among others. Starting December 2020, ZMC has offered the BNT162b2 mRNA-based vaccine to all its staff, including administrative and support staff (referred to as HCWs thereafter), with no specific exclusion for pregnant women. As at 21 January 2021, one-dose uptake was ca 90%.

Evidence from clinical trials has shown that immunogenicity translated to vaccine efficacy. Measuring circulating antibody levels enables the comparison of different vaccinated groups even though interpreting differences remains a challenge since there are no known correlates of protection for COVID-19 vaccines yet. Nevertheless, in order to describe one-dose immunogenicity among different groups, we measured anti-spike IgG levels against the severe acute respiratory coronavirus 2 (SARS-CoV-2) virus following administration of one dose of the BNT162b2 mRNA vaccine and reported them according to age, ethnicity, sex and prior infection status. Individuals with detectable IgG antibodies at baseline and/or evidence of a previous positive PCR test SARS-CoV-2 were considered previously infected.

## Measuring SARS-CoV-2 antibody levels before and after vaccination

Prior to vaccination, HCWs consented to their neutralising IgG antibody level being measured, using the Abbott Architect SARS-CoV-2 IgG qualitative assay (Abbott, Abbot Park, US), that detects nucleocapsid (N) IgG antibodies with high sensitivity and specificity [8]. Each positive sample was re-tested for verification purposes, using the quantitative LIAISON SARS-CoV-2 S1/S2 IgG assay (DiaSorin, Saluggia, Italy) [8]. In order to ascertain prior infection with SARS-CoV-2, we asked HCWs through a survey if they had a positive PCR test in the past and identified positive PCR tests for all participants in hospital records. HCWs were also asked to consent to IgG antibody level testing 21 days after dose 1 (at the same time as receiving dose 2), using the quantitative LIAISON Diasorin SARS-CoV-2 S1/S2 IgG assay, which unlike the Abbot assay is able to detect and quantify vaccine-induced antibodies [8].

The vaccine was offered to all HCWs regardless of consenting to antibody level measurement.

Antibody levels were reported using geometric mean concentration (GMC) alongside 95% confidence intervals (95% CI), stratified by age, ethnicity, sex and prior infection status. Trends in IgG levels by age were investigated using Cuzick non-parametric test for trend (an extension of the Wilcoxon rank-sum test). Among those with a previously positive PCR test, we determined association between time interval between a positive test and vaccination and post-vaccination IgG levels using linear regression. We compared the age and ethnicity of non-responders to others using Kruskall Wallis and chi-squared tests, respectively.

In order to visually display the range of antibody levels in a simpler way, we also described the distribution of IgG antibody levels using boxplots of the log10 IgG titres.

### Ethical statement

The study was approved by ZMC’s ethics committee (0133–20-ZIV).

## Impact of age, ethnicity, sex and prior infection on immunogenicity

Of the 1,378 HCWs who received the first dose of the vaccine, 514 (37%; age range: 19–77) took part in the study and had their antibody levels measured at 21 days. Among those who received the vaccine, 385 (74.9%) were tested for IgG levels at baseline, before vaccination. Of these, six were IgG positive including four HCWs who had evidence of a previously positive PCR test; an additional 11 HCWs had recorded evidence of a positive PCR test between March and November 2020 but were IgG negative. Among the 15 individuals with a previous positive PCR test, the time between positive PCR test and vaccination ranged between 39 and 308 days (median: 127 days).

Among all vaccinated HCWs, 475 (92%) had detectable anti-SARS-CoV-2 spike IgG antibodies and among these, GMC was 68.6 AU/mL (95% CI: 64–73.6). The 39 HCWs who did not respond to the first dose were older (median age 57 vs 45 in other, p < 0.001) and more likely to be Jewish (31/38 non-responders of known ethnicity, 82% vs 291/459 responders of known ethnicity; 63%; p = 0.01). Among responders, there was no statistically significant difference in antibody titres between males and females and between different ethnicities, but titres decreased with increasing age (p < 0.001, Table and Figure). The trend persisted when previously infected individuals were excluded (p < 0.001) (data not shown).

**Table:** Geometric mean concentration of anti-SARS-CoV-2 spike IgG antibodies among healthcare workers who responded to the BNT162b2 mRNA COVID-19 vaccine, 21 days post first dose, Israel, December 2020 to January 2021

Characteristics		Individuals in the sample (n=514)	Vaccine responders (n=475)	IgG geometric mean concentration among vaccine responders (AU/mL)^a^	95% CI
All participants with a detectable antibody response		475	475	68.6	64–73.6
Age (years)	< 30	11	10	100.4	51.8–194.5
	30–39	161	156	84.2	74.3–95.3
	40–49	146	139	68.2	60.2–77.4
	50–59	101	92	61.5	52.6–71.9
	60 +	95	78	49.8	42.6–58.1
Ethnicity	Jewish	322	291	62.4	58.2–66.9
	Arab	114	109	69.9	59.6–82
	Druze	58	57	73.4	58.6–92
	Circassian	2	1	–^b^	–^b^
	Missing	18	17	NA	NA
Sex	Male	193	177	64.6	60.2–69.2
	Female	321	298	75.9	65.6–87.9
Prior disease status^a^	All patients with evidence of prior COVID-19 infection	17	17	573.6	289–1,138.7
	IgG positive at baseline	6	6	747.3	140–3,978.3
	IgG negative with prior positive PCR test	11	11	496.5	217.4–1,134
	IgG negative at baseline and no prior positive PCR test	369	347	61.5	58–65.1
	Unknown (no PCR test and not tested at baseline)	128	111	64.3	60.5–68.3

CI: confidence interval; COVID-19: coronavirus disease; HCW: healthcare workers; SARS-CoV-2: severe acute respiratory coronavirus 2.

^a^ Geometric mean concentration calculation includes all HCWs who responded to the vaccine. Those with no detectable antibodies post-vaccination are excluded.

^b^ The GMC for Circassian HCWs is not given as only one HCW in this category responded to the vaccine.

**Figure:**
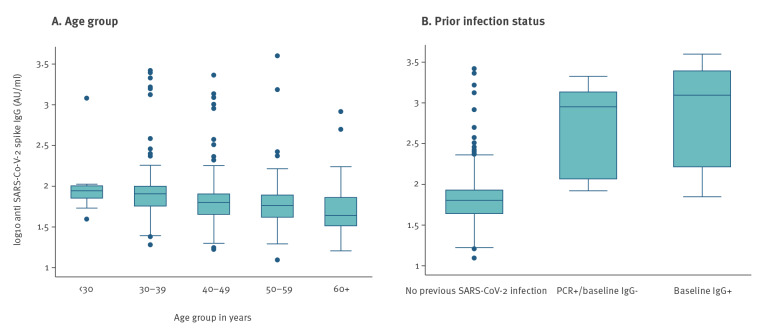
Anti SARS-CoV-2 spike IgG antibody levels among healthcare workers, 21 days post first-dose of BNT162b2 mRNA COVID19 vaccine by age (A) and prior infection status (B), Israel, December 2020 to January 2021 (n = 475)

COVID-19: coronavirus disease; SARS-CoV-2: severe acute respiratory coronavirus 2.

The boxplot displays the median value (line inside the box); the interquartile range (IQR) (25^th^ and 75^th^ centiles, represented by the box); the adjacent values (25^th^ centile-1.5*IQR and 75^th^ centile+1.5*IQR), and outlying values beyond adjacent values.

Compared with HCWs with no evidence of previous infection, post-vaccination IgG levels among those with previous evidence of infection were much higher (GMC 573 vs 61.5). IgG titres among those with previous evidence of infection were at least one order of magnitude higher than those without, regardless of whether IgG antibodies were detectable before being vaccination (Figure). Among individuals with a previously recorded positive PCR test, IgG levels post first dose of vaccine did not vary according to time from a positive test to vaccination (p = 0.165).

## Discussion

A single dose of the BNT162b2 mRNA COVID19 vaccine was immunogenic in the vast majority (92%) of our study cohort 21 days post vaccination, a result compatible with trial data [9]. Of note, our sample is relatively small and therefore did not allow for adjusted analyses. In addition, information on co-morbidities was not available and since the study population only includes HCWs, it may not be representative of the wider population: elderly individuals in particular are under-represented. Larger scale data should be analysed to confirm or refute such a possibility.

The differences in IgG levels by age found here are small and of unclear clinical significance in the absence of known correlates of protection. Efficacy data suggest similar efficacy among different age groups [3]. While our data suggest that age and ethnicity (but not sex) may be associated with the likelihood of non-response, these findings are based on 39 observations only. These associations should therefore be considered as hypothesis-generating and be tested using larger samples.

Unsurprisingly, vaccinating individuals with evidence of prior COVID-19 infection lead to a boost response, achieving IgG titres approximately one order of magnitude higher compared with naïve individuals. Interestingly, this was the case in our cohort regardless of whether SARS-CoV2 N antibodies were detectable or not immediately before vaccination and regardless of the time interval between infection and vaccination. Although these results are based on small numbers, i.e. 17 observations, they provide reassurance that the well documented rapid waning of nucleocapsid IgG antibodies post-acute COVID-19 infection [10] does not necessarily translate to a loss of immunity. The boost-like response seen among previously infected individuals in our cohort suggests B-cell-mediated memory immunity is preserved regardless of IgG status. Our study confirms recently published evidence suggesting that immune memory persists at least 6 months post infection [11]. One single case in our cohort who showed a boost-type response almost 10 months after testing positive by PCR suggests this could be even longer. In situations of scarce vaccine availability, it may therefore be possible to assume that most individuals with prior evidence of infection are not prioritised for vaccination, regardless of pre-vaccination IgG levels. Nevertheless, infection does not protect 100% against a re-infection [12] and offering vaccination to these individuals may confer additional protection, as major public health agencies recommend [13]. A single dose of vaccine in these individuals seems to boost the response although the optimal timing between infection and vaccination as well as the ensuing duration of protection remain to be determined.

Our study only contains a small number of previously infected individuals, as the Israeli ministry of health guidelines recommended that these individuals are not prioritised for vaccination, and findings from this study should be replicated on a larger scale in order to make policy decisions. As the immunisation programme continues to expand in Israel, previously infected HCWs will be offered the vaccine and we will continue to assess antibody levels in these HCWs as well as in all others following the administration of the second dose of vaccine. Trends in antibody response following two doses of vaccine among the different groups of HCWs that comprise ZMC’s workforce are also being analysed and will be shared as they become available.
